# P-1541. Antimicrobial susceptibility and antimicrobial synergy testing of human *Streptococcus suis* isolates in Thailand

**DOI:** 10.1093/ofid/ofae631.1708

**Published:** 2025-01-29

**Authors:** Wilawan Thipmontree, Rattagan Kajeekul, Athita Lewrod, Anusak Kerdsin, Suganya Yongkiettrakul

**Affiliations:** Maharat Nakhon Ratchasima Hospital, Muang, Nakhon Ratchasima, Thailand; Maharat Nakhon Ratchasima Hospital, Nakhon Ratchasima, Nakhon Ratchasima, Thailand; Maharat Nakhob Ratchasima Hospital, Amphur Muang, Nakhon Ratchasima, Thailand; Kasetsart Unuversity, Muang, Sakon Nakhon, Thailand; National Science and Development Agency (NSTDA), Klong Lunag, Pathum Thani, Thailand

## Abstract

**Background:**

*Streptococcus suis* (*S. suis*) infection can cause serious infections with high morbidity and mortality. Both humans and pigs have reported emerging antimicrobial resistance. This study aims to investigate the antimicrobial susceptibility test (AST) and antimicrobial synergy testing in *S. suis* human isolates.

**Table 1.**
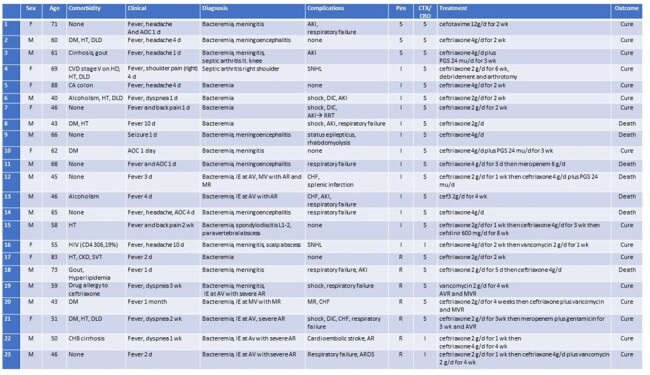
Clinical manifestations of 25 culture-confirmed S. suis infection cases who performed an antimicrobial synergy test.

**Methods:**

We conducted a retrospective study of patients with culture-confirmed *S. suis* infection in Northeast Thailand. We analyzed the clinical manifestations, the AST and selected 23 isolates for determining the antimicrobial synergy effect via checkerboard microdilution.

**Table 2:**
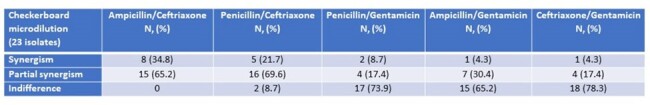
Antimicrobial synergy testing by checkerboard microdilution in 23 S. suis human isolates.

**Results:**

Between 2021 and 2023, we included 253 culture-confirmed *S. suis* patients; the mortality rate was 15%. A total of 194 patients underwent echocardiograms, and 95 (48.9%) patients had infective endocarditis. The prevalence of *S. suis* strain resistance to penicillin increased from 7.8% in 2021 to 31.9% in 2023. Additionally, the intermediate susceptibility of *S. suis* to CRO/CTX rose from 3.1% in 2021 to 9.9% in 2023. Moreover, in 2023, two isolates of *S. suis* showed resistance to CRO/CTX.

We performed antimicrobial synergy testing in response to the surge in antimicrobial resistance of *S. suis* infection. The AST of 23 isolates exhibited the following: susceptible to both penicillin and ceftriaxone/cefotaxime (CRO/CTX) (3); intermediately sensitive to penicillin but sensitivity to CRO/CTX (12); intermediately sensitive to both penicillin and CRO/CTX (1); resistance to penicillin but sensitive to CRO/CTX (5); and resistance to penicillin but intermediately sensitive to CRO/CTX (2).The combination of ampicillin and ceftriaxone demonstrated synergy in 34.8% (8/23) of cases, with 65.2% showing a partial synergism effect. Penicillin and ceftriaxone showed synergy in 21.7%, partial synergy in 69.6%, and indifference in 8.7%. Conversely, the results for ampicillin-gentamicin, penicillin-gentamicin, and ceftriaxone-gentamicin showed mostly indifference: 65.2%, 73.9%, and 78.3%, respectively.

**Conclusion:**

*S. suis* infection is associated with high mortality rates and increased resistance to antimicrobials. A combination therapy comprising ceftriaxone and ampicillin may be the optimal and most effective treatment option.

**Disclosures:**

**All Authors**: No reported disclosures

